# Machine learning predicts distinct biotypes of amyotrophic lateral sclerosis

**DOI:** 10.1038/s41431-025-01920-y

**Published:** 2025-08-07

**Authors:** Nicholas Pasternack, Ole Paulsen, Avindra Nath

**Affiliations:** 1 Section of Infections of the Nervous System, National Institute of Neurological Disorders and Stroke (NINDS), National Institutes of Health (NIH), Bethesda, MD USA; 2 Department of Physiology, Development and Neuroscience, University of Cambridge, Cambridge, UK

**Keywords:** Genome informatics, Diseases of the nervous system, Gene regulatory networks, Genetics of the nervous system

## Abstract

Amyotrophic lateral sclerosis (ALS) is a neurodegenerative disease that is universally fatal and has no cure. Heterogeneity of clinical presentation, disease onset, and proposed pathological mechanisms are key reasons why developing impactful therapies for ALS has been challenging. Here we analyzed data from two postmortem cohorts: one with bulk transcriptomes from 297 ALS patients and a separate cohort of single cell transcriptomes from 23 ALS patients. Using unsupervised machine learning, we found three groups of ALS patients characterized by synaptic dysfunction (34%), neuronal regeneration (47%), and neuronal degeneration (19%). Each of these ALS subtypes had unique patterns of transcriptional dysregulation that could represent novel therapeutic targets. We then developed a supervised machine learning model that was about 80% accurate at predicting ALS subtype based on patient demographic and clinical data. Together, we established three biologically distinct subtypes of ALS that can be predicted by clinical and demographic data.

## Introduction

Amyotrophic lateral sclerosis (ALS) or Motor Neurone Disease is a fatal neurodegenerative disease that results in the degeneration of both upper motor neurons and lower motor neurons, with a usual age of onset between 50 and 65 years [1]. ALS patients have a heterogeneous presentation, with multiple underlying pathophysiological mechanisms, which make developing impactful disease course-modifying therapies and reliable biomarkers challenging. This is further complicated by the substantial number of genetic mutations that have been implicated in the disease, which have their own unique pathophysiology. However, most cases are sporadic. Hence, identifying biotypes of ALS based on underlying pathophysiological mechanisms may be key to developing diagnostics and targeted therapies [2, 3]. Past attempts to classify ALS based on site of onset of motor symptoms, rate of progression, age of onset, and other demographic features have some prognostic value but have failed to elucidate distinct pathophysiological mechanisms. Recent developments in RNA sequencing (RNA-seq) and bioinformatics have started to provide unique insight into the pathophysiology of ALS and the heterogeneity associated with it. Using this approach, one study described three transcriptionally distinct subpopulations of ALS characterized by oxidative stress, glial cell activation, or transposable element activation [4].

We used machine learning tools to identify clusters of ALS patients that display similar pathophysiological processes based on RNA-seq. This allowed us to process data from a large sample size and do so in an unbiased manner. We included additional tools to analyze endogenous retroviral transcripts since they have been previously implicated in the pathophysiology of ALS [5–10]. Furthermore, we analyzed both cortical and spinal cord samples, as well as ALS patients only and ALS patients and unaffected controls. Using these approaches, we have defined three pathophysiologically distinct subtypes of ALS which relate to synaptic dysfunction, neuronal regeneration, and neuronal degeneration. We determined the relevance of these clusters to biological pathways, cell types, and transcriptional regulators of interest. This has allowed us to identify potential therapeutic targets unique to the three biotypes of ALS. Finally, we developed a machine learning classifier that was about 80% accurate at determining whether individuals belonged to a given non-negative matrix factorization (NMF) cluster based on easily obtained phenotypic information.

## Results

### Unsupervised clustering reveals three transcriptionally distinct ALS patient populations

We used NMF to determine if the ALS patient cohort could be grouped into clusters based on their transcriptional profiles. Sex-associated features were filtered out prior to clustering. Clustering was then confirmed via principal component analysis (PCA) and complete linkage hierarchical clustering with Euclidean distance function using the “aheatmap” function of the NMF library in R. Filtering out features associated with biological sex removed the influence of sex on clustering (Supplementary Fig. 9).

For the first set of analyses, we analyzed ALS patients only (ALS) and analyzed the CTX and SC samples separately. We discovered three transcriptionally distinct clusters of ALS patients in the CTX and the SC groups (Supplementary Fig. 9A, B). Of these clusters, CTX_ALS_2 was the largest cluster in the CTX with 47% of ALS samples, and SC_ALS_3 was the largest cluster in the SC with 48% of ALS samples. Next, we combined ALS and controls (ALSC) and reanalyzed by NMF to determine if the clustering pattern of the ALS analysis was robust. Four clusters were identified, of which three were enriched with ALS patients, with CTX_ALSC_4 and SC_ALSC_2 each comprising 48% of the ALS and control samples, with over 80% of the samples coming from the ALS cohort (Supplementary Fig. 9C, D; Supplementary Table 4). To identify distinguishing features between the clusters, we first analyzed them for demographic features. A one-way ANOVA for each of the four NMF analysis types with subsequent Tukey’s Honest Significant Difference test showed no significant difference in terms of age at death between any of the ALS and patient-enriched ALSC clusters. However, there were significant differences between the ALSC control and patient-enriched clusters (*p*-adj < 0.01). A Kruskal–Wallis test revealed no significant differences in terms of biological sex between clusters for each of the four analyses. Finally, a Kruskal–Wallis test followed by pairwise FDR-adjusted Wilcoxon tests revealed significant differences (*p*-adj < 0.05) between SC_ALSC_3 and the other three SC_ALSC clusters in terms of *C9orf72* positivity. The features that distinguish the ALS clusters from each other (i.e., selected features) were more similar in the ALS than in the ALSC analysis: only 4.6% of NMF selected features were shared between CTX and SC ALSC analyses, whereas 61% of selected features were shared between CTX and SC ALS analyses. Furthermore, the shared selected features were significantly correlated with each other in the ALS analyses but not the ALSC analyses (Supplementary Fig. 3).

### Analysis of cluster-specific HERV-K dysregulation

We next determined if there were significant differences in terms of expression of HERV-K features between NMF groups. Although no HERV-K protein coding features were part of the selected features group, the NMF algorithm used for the determination of cluster types, there was one HERV-K feature in cluster CTX_ALSC_1, and two features in CTX_ALSC_2 that distinguish them from the other clusters (Supplementary Data 1–4).

We next performed differential expression analysis (DEA) comparing each NMF cluster with all other clusters in each group (e.g., CTX_ALS_1 vs CTX_ALS_2 and CTX_ALS_3). This showed that individual HERVs were differentially expressed between clusters (Supplementary Figs. 4–7). However, no clear patterns or differences at the gene set level among patient clusters were identified (Supplementary Fig. 8). The only significant increase in HERV-K expression at the gene set level was in SC_ALSC_2. However, there were five HERV-K proviruses (3q21.2, 6q14.1, 8p23.1, 19q13.42, and 19q11) encoding a full-length Env that were upregulated in at least one of the 14 DEAs (Supplementary Table 1). No NMF cluster showed increased expression of more than three of these loci. Only HERV-K proviruses 3q21.2 and 19q11 were upregulated in a cluster in each of the four NMF analyses: 3q21.2 was upregulated in CTX_ALS_2, CTX_ALSC_1, SC_ALS_3, SC_ALSC_2, while 19q11 was upregulated in CTX_ALS_1, CTX_ALSC_2, SC_ALS_1, and SC_ALSC_2. SC_ALSC_2, the largest and second most patient-enriched cluster for that analysis type, had upregulation of 3q21.2, 8p23.1, 19q11 (the most of any cluster). None of the non-patient-enriched clusters (ALSC_CTX_3 and ALSC_SC_4) exhibited upregulation of a HERV-K Env-coding feature. Thus, expression of specific HERV-K loci was increased in ALS patient subpopulations.

### Pathway analysis of differentially expressed genes distinguishes the clusters identified by NMF

**Fig. 1:**
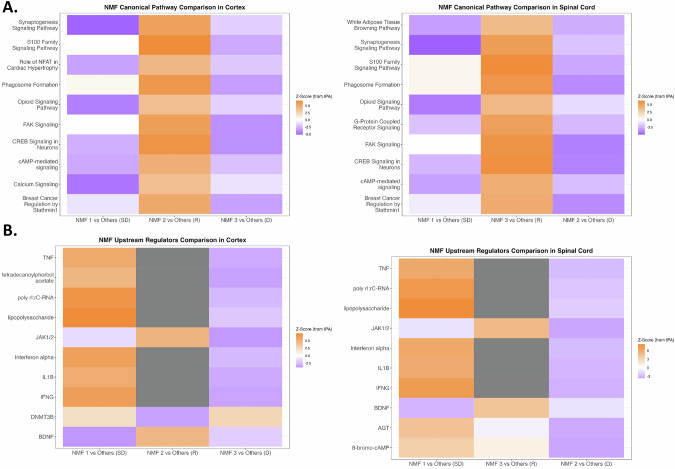
IPA canonical pathways and upstream regulators analysis for NMF clusters derived from ALS patients. IPA results for canonical pathway analysis (**A**) and upstream regulator analysis (**B**) using DEA results comparing each NMF cluster with the other clusters in the cortex or spinal cord. The heatmaps are ordered by and list the pathophysiological category of the cluster (synaptic dysfunction (SD), neuronal regeneration (R), or neuronal degeneration (D)) is also listed. Z-score values were exported from IPA. Values > |2| are significant. Orange boxes represent upregulated features, blue boxes represent downregulated features, and gray boxes represent undetermined.

To further determine the gene expression patterns unique to each of the ALS clusters, we compared the transcriptional pattern of each of the clusters to the other two in the CTX or SC groups using ingenuity pathway analysis (IPA). IPA is a type of pathway analysis that utilizes a curated knowledgebase and standardized analysis approach to analyze the broader biological relevance of gene expression data. The top ten most dysregulated IPA pathways (based on absolute value of the Z-score) across cluster-specific DEAs were selected for further comparison. When CTX_ALS_1 was compared to CTX_ALS_2 and CTX_ALS_3, it showed downregulation of synaptogenesis pathways, which was associated with decreased opioid and calcium signaling pathways. However, there were minimal differences in terms of the S100, phagosome, and stathmin pathways. This suggests that this cluster is enriched with individuals exhibiting primarily synaptic dysfunction. In contrast, CTX_ALS_2 had strong activation of all these pathways, which is suggestive of ongoing regeneration, and CTX_ALS_3 had downregulation of these pathways, suggestive of degeneration. A similar pattern was noticed in the spinal cord, where the transcriptional pattern of SC_ALS_1 resembled that of CTX_ALS_1 and was thus representative of synaptic dysfunction. Here, cluster SC_ALS_2 showed a pattern consistent with degeneration and SC_ALS_3 with regeneration (Fig. 1A). We next used IPA to identify the upstream regulators that may be responsible for gene expression changes in each of the clusters. This analysis identifies the predicted activation or inhibition state of molecules that can regulate the expression of the transcriptional pathways dysregulated in the clusters. CTX_ALS_1 and SC_ALS_1 were predicted to have inhibition of BDNF- and JAK1/2-mediated pathways, consistent with the pattern of synaptic dysfunction. These clusters exhibited transcriptional patterns consistent with activation of interferon alpha and gamma, IL1β, and TNF, as well as lipopolysaccharide, poly-IC, and tetradecanoylphorbol acetate, which suggests associated glial cell activation. In contrast, CTX_ALS_2 and SC_ALS_3 showed activation of BDNF and JAK1/2 regulated pathways and only a mild activation of the aforementioned glial cell pathways. This pattern is consistent with regeneration. CTX_ALS_3 and SC_ALS_2 showed predicted inhibition of all upstream regulators, which is consistent with degeneration (Fig. 1B).

**Fig. 2:**
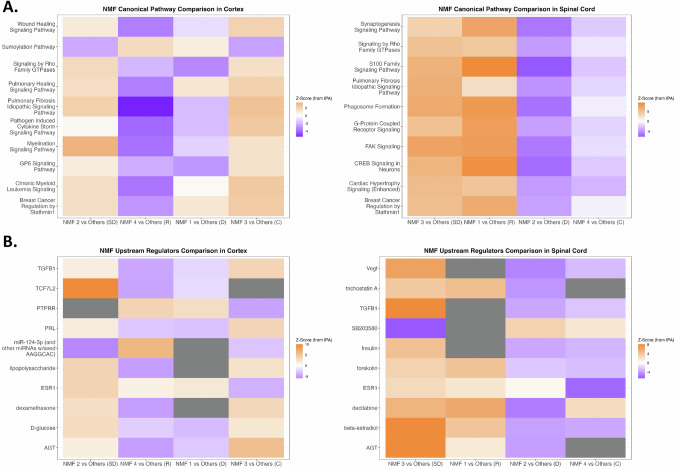
IPA canonical pathways and upstream regulators analysis for NMF clusters derived from both ALS patients and control samples (ALSC). IPA results for canonical pathway analysis (**A**) and upstream regulator analysis (**B**) using DEA results comparing each NMF cluster with the other clusters in the cortex or spinal cord. The heatmaps are ordered by and list the pathophysiological category of the cluster (synaptic dysfunction (SD), neuronal regeneration (R), neuronal degeneration (D), or unaffected control (C)) is also listed. Z-score values were exported from IPA. Values > |2| are significant. Orange boxes represent upregulated features, blue boxes represent downregulated features, and gray boxes represent undetermined.

**Fig. 3:**
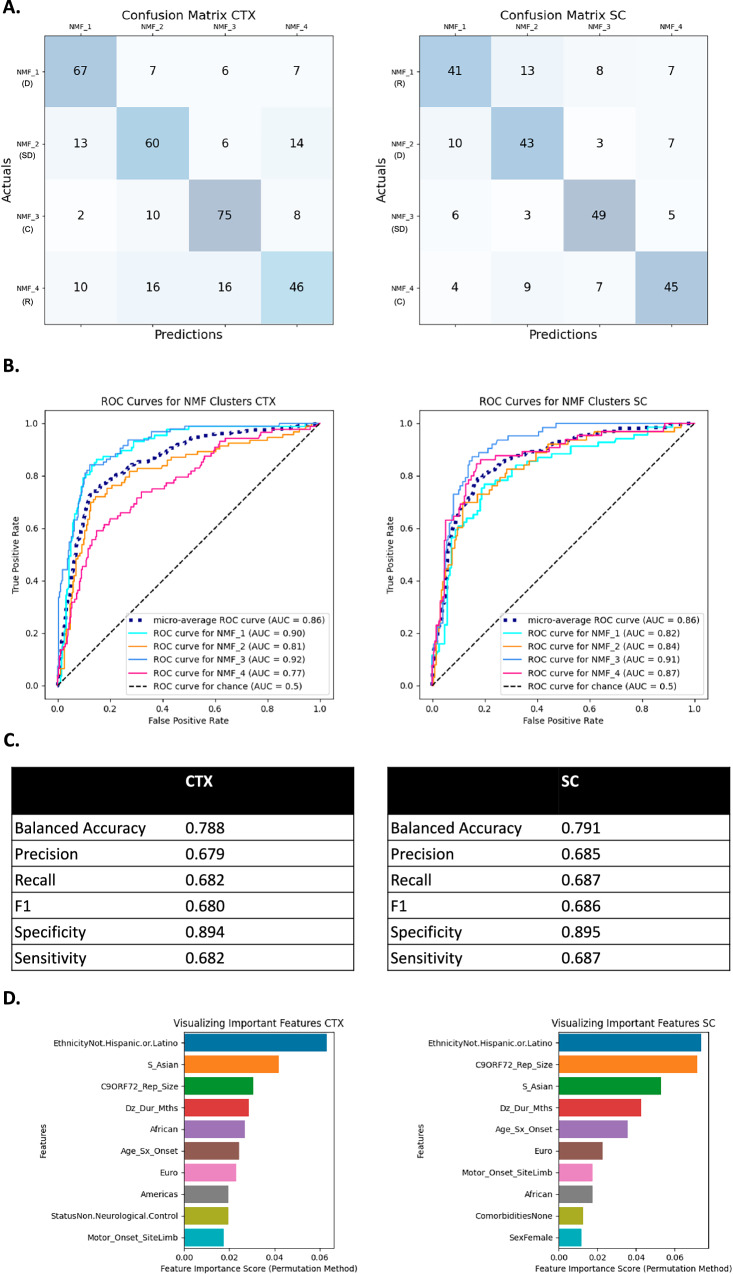
Summary of random forest classifier (RFC) performance for identifying non-negative matrix factorization (NMF) cluster in ALS patients. One verse rest (OvR) RFC results from cortex (CTX) and spinal cord (SC) analyses. Confusion matrices, corresponding to predicted NMF cluster (columns) and actual cluster (rows) (**A**); ROC curves, true vs false positive rates plotted along with the AUC with different style lines for the four NMF classifiers along with a micro-average of the four (dotted blue line) and a dotted black line for chance-based performance (**B**); selected model performance metrics (**C**); and a bar plot of the top 10 most important features along with their permutation importance scores (**D**) for RFC-based models in the CTX (left) and SC (right). Under the NMF ID, the pathophysiological relevance of the cluster is indicated by a R (neuronal regeneration), SD (synaptic dysfunction), D (neuronal degeneration), or C (control).

We next performed a similar analysis in the ALSC cohort to determine if the findings from the ALS cohort could be validated in the clusters of this cohort. The top ten pathways dysregulated in the ALSC_CTX samples involved cellular repair, cytokine signaling, protein degradation, and stathmin pathways. The pathways dysregulated in the ALSC_SC showed substantial overlap with those involved in the ALS_SC samples (Figs. 2A and 3A). The ALSC cohort had four clusters, of which clusters CTX_ALSC_3 and SC_ALSC_4 contained a substantial percentage of control samples. CTX_ALS_2 and SC_ALS_1 showed upregulation of most pathways and hence may be suggestive of regeneration. CTX_ALSC_4 and SC_ALSC_2 showed downregulation of most pathways and hence are suggestive of degeneration. CTX_ALS_1 showed downregulation of Rho-GTPase and GP6 signaling pathways and upregulation of cellular repair pathways and stathmin pathways. SC_ALSC_3 showed a relative decrease in synaptogenesis pathway and upregulation of several secondary signaling pathways. Hence, these patterns may be consistent with synaptic dysfunction (Fig. 2A). Upstream regulator analysis showed dysregulation of cytokine pathways, steroid-regulated, and steroid/insulin-regulated pathways. Additionally, in the SC, there was dysregulation of vascular endothelial growth factor, and trichostatin A regulated histone deacetylation pathways. Again, three clusters in the ALSC_CTX and ALSC_SC enriched in ALS samples could be distinguished from each other based on predominant downregulation suggestive of degeneration, predominant upregulation suggestive of regeneration, and a mixed pattern suggestive of synaptic dysfunction (Fig. 2B).

Thus, the NMF clusters in cortex and spinal cord show that in both anatomical regions, there are three broad groups of clusters. These clusters can be distinguished based on the pattern of biological pathways dysregulated and may represent different stages of neuronal degeneration or repair. The neuronal regeneration (CTX_ALS_2 and SC_ALS_3) group also represents the largest cluster and had the highest percentage of samples positive for *C9orf72* mutations. The group with predominantly synaptic dysfunction was an intermediate-size group, and the third group, with evidence of neuronal degeneration, represents the smallest group (Fig. 1A). The synaptic loss may be driven by activation of cytokine and interferon pathways and loss of BDNF. The clusters with neuronal regeneration also showed activation of BDNF while the neuronal degeneration clusters had loss of all signaling pathways (Fig. 1B).

### NMF clusters can be predicted using machine learning trained on phenotypic data

To determine the feasibility of predicting an individual’s NMF cluster based on commonly collected phenotypic and demographic data, a random forest classifier (RFC) was developed using the metadata provided by NYGC along with the NMF designations determined in this study. Prior to the analysis, the data was split into training (80%) and testing (20%) sets.

The classifier for the ALS analysis had an average balanced accuracy of 69% across the CTX (65% accurate) and SC (72% accurate) (Fig. 3C) and was more specific than sensitive across the CTX and SC. The classifier was most accurate for the neurodegeneration clusters, CTX_ALS_3 and SC_ALS_2, which were also the smallest clusters (Fig. 3A, B). The average AUC was 0.7 for the CTX and 0.77 for the SC (Fig. 3B), indicating the model fit the data well. The age of symptom onset, *C9orf72* repeat size, and ethnicity were the most important features to the model (Fig. 3D).

**Fig. 4:**
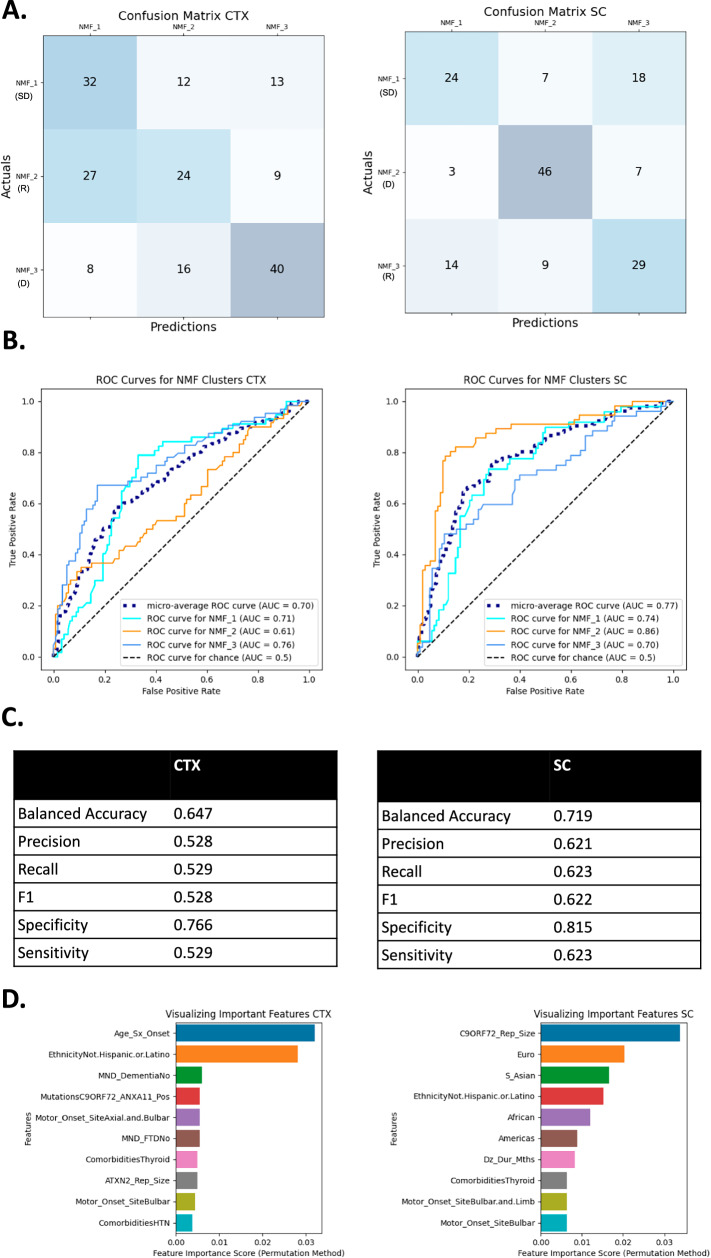
Summary of random forest classifier (RFC) performance for identifying non-negative matrix factorization (NMF) cluster in both ALS patients and controls. One verse rest (OvR) RFC results from cortex (CTX) and spinal cord (SC) analyses. Confusion matrices, corresponding to predicted NMF cluster (columns) and actual cluster (rows) (**A**); Receiver operating characteristic (ROC) curves, true vs false positive rates plotted along with the area under the curve (AUC) with different style lines for the four NMF classifiers along with a micro-average of the four (dotted blue line) and a dotted black line for chance-based performance (**B**); selected model performance metrics (**C**); and a bar plot of the top 10 most important features along with their permutation importance scores (**D**) for RFC-based models in the CTX (left) and SC (right). Under the NMF ID, the pathophysiological relevance of the cluster is indicated by a R (neuronal regeneration), SD (synaptic dysfunction), D (neuronal degeneration), or C (control).

This analysis was repeated with the ALSC cohort. The average balanced accuracy was greater than for the ALS cohort: 79% for CTX. The accuracy for SC was also 79% (Fig. 4C). In CTX, CTX_ALSC_1, a neuronal degeneration cluster, and CTX_ALSC_3, the control cluster, were the most accurately predicted NMFs – both are smaller and less patient-enriched clusters. In SC, SC_ALSC_2 (largest cluster, highly enriched for patients, and a neuronal degeneration cluster) and SC_ALSC_4 (small and a control cluster) were the most accurately predicted (Fig. 2A). The number of false positives and negatives made by the classifier were similar (Fig. 4C). Additionally, the average area under the curve (AUC) of the receiver operating characteristic (ROC) curve across all four NMF’s was 0.86 in both the CTX and SC. Thus, while the model was better at predicting certain NMF clusters, it generally performed similarly in CTX and SC, was accurate, and fit the data well. Ethnicity, *C9orf72* repeat size, and disease duration were the most important features for the model (Fig. 4D).

Thus, clusters with pathophysiologies related to neuronal degeneration and control clusters were the most accurately predicted by our classifier across all four NMF analyses.

### Analysis of cluster-specific HERV-K dysregulation

When we looked for Env expressing HERV-K loci in the clusters that were grouped based on the three pathophysiological patterns described above, a consistent pattern emerged. Loci 8p23.1 and 19q11 were upregulated only in the synaptic dysfunction biotype in the cortex, and in the synaptic dysfunction or neuronal degeneration biotypes in the spinal cord. Locus 19q13.42 was upregulated in three synaptic dysfunction clusters (all CTX and SC ALS only analyses). Loci 6q14.1 were upregulated in a neuronal regeneration cluster (CTX_ALS_2), and 3q21.2 was upregulated in neuronal regeneration and degeneration clusters. Moreover, none of the non-patient-enriched clusters (ALSC_CTX_3 and ALSC_SC_4) exhibited upregulation of a HERV-K Env-coding feature (Supplementary Table 1). Thus, expression of specific Env-coding HERV-K loci was upregulated in a pathophysiological cluster-dependent fashion.

### NMF clusters represent subgroups of patients with different cellular pathophysiologies

**Fig. 5:**
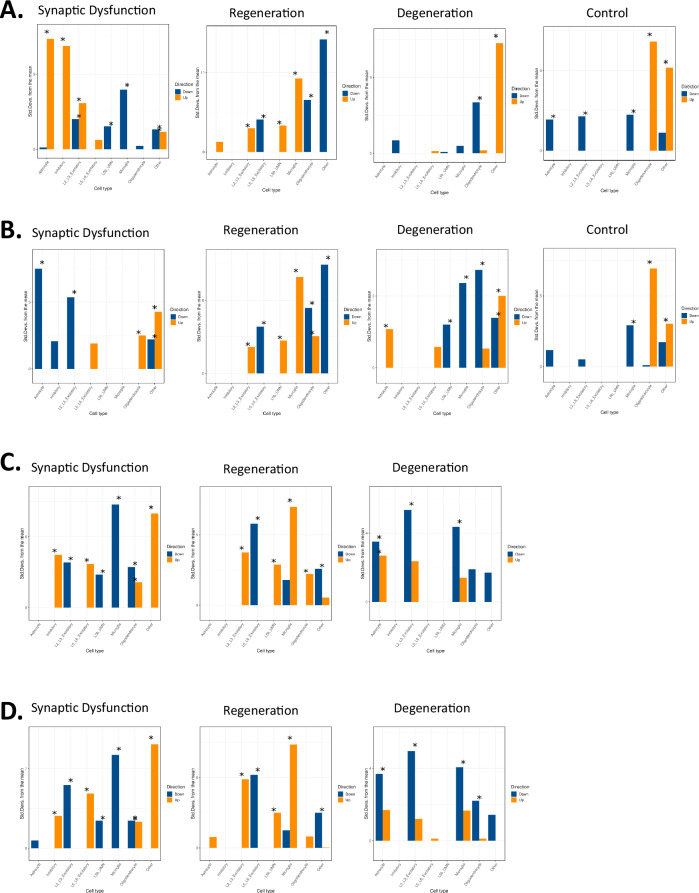
Estimated cell enrichments for NMF clusters in the cortex and spinal cord. Standard deviations (SDs) from mean based on empirical null distribution are plotted for cortex (CTX) and spinal cord (SC) samples using Expression Weighted Cell type Enrichment (EWCE). Results are plotted for both ALS patients and controls (ALSC) in the CTX (**A**), and SC (**B**). As well as the ALS patients, only NMF analysis for CTX (**C**) and SC (**D**). NMF clusters are ordered according to their pathophysiology (synaptic dysfunction, neuronal regeneration, neuronal degeneration, or control). Asterisks represent significant effects based on Bonferroni corrected p-value. The color of the bar represents whether the analysis was performed on the 250 most upregulated (orange) or downregulated (blue) features from the corresponding DEA. Only positive differences of SDs from mean are shown. Each cluster had a distinct pattern of estimated cell group-specific up- or downregulation.

Using a publicly available scRNA-seq dataset from cortical samples of both ALS and control samples [11], we determined whether the transcriptional signatures associated with each cluster could represent different cellular pathologies via Expression Weighted Cell type Enrichment (EWCE) [12] (Fig. 5). Based on this analysis, as well as the IPA (Figs. 2 and 3), we determined that the three distinct groups of ALS patients we identified in both CTX and SC had three distinct pathophysiologies representing a range from neuronal regeneration to degeneration: neuronal regeneration (47%), synaptic dysfunction (34%), and neuronal degeneration (19%). Like the IPA, in the EWCE analysis, the ALS clusters were more similar across CTX and SC than the ALSC clusters.

The neuronal regeneration clusters (CTX_ALS_2 and SC_ALS_3) from the ALS analyses exhibited upregulation of excitatory neuron-, upper motor neuron-, and microglia-related genes. The synaptic dysfunction clusters (CTX_ALS_1 and SC_ALS_1) had upregulation of inhibitory neuron- and downregulation of upper motor neuron- and excitatory neuron-related genes. The neuronal degeneration clusters (CTX_ALS_3 and SC_ALS_2) had downregulation of astrocyte-, microglia-, and excitatory neuron-related genes.

This pattern of cellular dysregulation was also seen in the ALSC cohort. CTX_ALSC_4 and SC_ALSC_1, representing the neuronal regeneration clusters, exhibited upregulation of excitatory neurons and upper motor neuron genes, which was accompanied by microglial cell activation. CTX_ALSC_2 and SC_ALSC_3, which represent the synaptic dysfunction clusters, had a mixed pattern of up and downregulation of excitatory neuron and upper motor neuron-related genes. CTX_ALSC_1 and SC_ALSC_2, representing the neuronal degeneration clusters, showed downregulation of most cell types with the exception of astrocyte activation in the SC.

## Discussion

Previous studies have used NMF to identify transcriptionally distinct subgroups using either motor or frontal cortex samples from ALS patients only [4, 13]. Benefits of our study include examining the robustness of unsupervised clustering in the cortex and the spinal cord, and with the addition of unaffected control samples. Additionally, our pathway analysis strategy also enabled us to highlight key transcriptional regulators that may mediate progression of ALS. By integrating endogenous retrovirus elements into our analysis, we were able to make predictions about which specific loci may be important mediators of disease. Finally, our use of supervised machine learning demonstrated that specific clinical and demographic characteristics can be used to predict ALS subtypes.

Limitations of this study include the fact that postmortem tissues mostly represent late-stage disease. Some samples, particularly in the ALSC analysis, likely represent transcriptionally intermediate cases that do not fall neatly into an NMF group. Future studies might use an ensemble clustering method by combining NMF with other clustering methods such as hierarchical clustering or graph-based clustering algorithms. Additionally, the different pathophysiological subgroups may be relevant at different times during the disease course. Models that take this into account may be able to optimize the timing of therapeutic interventions. Our three-subgroup model of ALS is likely a simplification of the underlying pathophysiology. While the three-subgroup model fits our RNA-seq data well and concurs with previous studies examining similar datasets, future studies might focus on algorithms that generate more subgroups with fewer individuals.

The synaptic dysfunction clusters were characterized by downregulation of synaptic signaling pathways. However, several secondary signaling and cell repair pathways were activated. This is also supported by cell-type analysis, which shows activation of neuronal transcripts. Histopathological studies in ALS patients have also shown variable amounts of loss of synapses in the anterior horn of the spinal cord. The amount of synaptic loss was predicted by disease duration, clinical site of onset, and loss of α-motoneurons [14]. The central role of synaptic dysfunction in ALS pathogenesis has been demonstrated at the transcriptomic, epigenetic, and alternative splicing levels in ALS motor neuron models and patient-derived blood and spinal cord samples [15, 16]. Therapeutic approaches targeting neuronal survival and synaptogenesis may be reasonable approaches for these patients.

The neuronal regeneration clusters were characterized by upregulation of pathways including secondary signaling pathways, protein degradation, cytokine regulation, neurotrophic factors, and cell repair. Cellular analysis showed a predominance of both neuronal and glial cell activation. Upregulation of immune genes and glial cell activation have been well studied in ALS [17]. Most studies have been focused on neuronal degeneration, and the possibility of ongoing neuronal regeneration has received less attention. However, this is not a surprising observation. The pattern of neurodegeneration in ALS is cell-type specific [18]. The neurons that are spared sprout axons and reinnervate the targets of the degenerating neurons. This phenomenon has been well studied in the spinal cord, and fiber type grouping and excitation of motor units are the hallmark of ALS [19]. Nearly half the ALS patient population was part of the neuronal regeneration group. This is in line with some previous studies. For example, regeneration of CNS cells was demonstrated in a cell culture-based motor neuron model of ALS and in a postmortem histological study of the spinal cord [20, 21]. Thus, therapeutic approaches targeting the neuro-glial interactions and facilitation of the neuronal regenerative processes could be considered in this population.

The third cluster had downregulation of most genes and pathways analyzed. Cell-type analysis showed downregulation in neurons and glia with some astrocytosis. These findings are most consistent with a degenerative process resulting in loss of neurons and the absence of a glial reaction. This likely represents end-stage disease at which point most therapeutic interventions are unlikely to alter the disease course. It is possible that these processes may be a continuum, and synaptic dysfunction may be an early event in the pathophysiology of ALS even though some patients exhibit these findings at the time of death. Patients may then transition to either a degeneration or a regeneration phase (Supplementary Fig. 10).

Pathways mediated by brain-derived neurotrophic factor (BDNF) and Janus Kinase 1/2 (JAK1/2) were key differentiators between the neuronal degeneration and regeneration ALS biotypes across CTX and SC. Meanwhile, SB203580, a mitogen-activated protein kinase (MAPK) inhibitor; Transcription factor 7-like 2 (TCF7L2); and Estrogen Receptor 1 (ESR1) differentiated the controls from the synaptic dysfunction biotype in the ALSC analysis. ESR1 was important across CTX and SC, while TCF7L2 was more CTX-specific and MAPK was more SC specific. BDNF, a neuronal growth factor, has been well studied in ALS. However, it can be both neuroprotective and detrimental to motor neurons in models of ALS depending on associated factors such as excitotoxicity [22]. SB203580 inhibited SOD1-mediated cell death of motor neurons in a mouse model of ALS [23]. Additionally, p38 MAPK inhibitors restored axonal transport deficits early in disease in a SOD1 mouse model of ALS [24]. Finally, estradiol has been implicated as a protective factor in ALS [25].

Our upstream regulatory analysis identified several molecules that regulate pathways dysregulated in ALS. Some of these molecules may have therapeutic potential. Decitabine reduced *C9orf72* repeat-expanded RNAs in experimental models [26]. Importantly, transcriptional pathways involved with decitabine were inhibited specifically in SC_ALSC_3, which had the highest percent C9orf72-positive patients. Transforming growth factor beta 1 (TGFB1) was downregulated in ALS-enriched clusters in brain and spinal cord. The TGFB1 pathways consist of cytokines involved in multiple functions, especially during development. TGFB1 was increased in the CSF and/or serum of ALS [27] in some but not all studies, which is indicative of the heterogeneity within this population. Angiotensinogen (AGT), a proteinase inhibitor involved in the renin-angiotensin system, exhibited a similar pattern to TGFB1. Angiotensin-converting enzyme inhibitors have already been shown to reduce the risk of ALS in animal models and in a case-control study [28]. Genes related to the opioid signaling pathway were dysregulated in the brain and spinal cord. A mutation in the sigma receptor type 1 (S1R) gene can cause early onset ALS [29], and the S1R has been shown to be neuroprotective in mouse models of ALS [30, 31].

Previous studies have implicated HERV-K in the pathophysiology of ALS [5–10]. Here we found that several loci that encode for HERV-K were dysregulated in the cortical and spinal cord tissues of specific groups of ALS patients. HERV-K Env-coding locus 19q13.42 was only upregulated in samples exhibiting neuronal degeneration. Therefore, especially if these pathophysiological categories represent a continuum, silencing this locus may represent an important therapeutic target to alter the progression of ALS.

Utilizing supervised machine learning, we accurately predicted NMF biotype. The most important factors for our model overall involved ethnicity and disease duration characteristics: both previously implicated in ALS [32, 33]. The machine learning model we developed, or a similar approach, could be used to inform future clinical trials to address the issue of ALS patient heterogeneity and develop treatment modalities that specifically target the different biotypes of ALS.

## Materials and methods

### Patients and samples

Details of the dataset, including sample preparation, metadata information, and our computational approach for analyzing endogenous retrovirus (ERV) loci, have been previously described [34]. There were 996 cortical (CTX) samples from 439 individuals and 714 spinal cord (SC) samples from 354 patients. Overall, 46% of the samples were female and 54% were male. In all, 297 ALS patients contributed 1250 samples and 179 controls contributed 460 samples. We used TEtranscripts [35] to measure ERV feature counts in addition to a standard transcript enumeration analysis pipeline. All patient samples were de-identified and were collected according to the appropriate ethical standards for each participating institution.

Across all SC and CTX samples, the average RNA integrity number (RIN) was 6 (standard deviation = 1.5) and the average postmortem interval (PMI) was 25 h (standard deviation = 25 h); the average age at death across all samples was 65 years (standard deviation = 12) and the average age at symptom onset was 61 years (standard deviation = 11). Limb onset was the most common site of motor symptom onset (62%), and *C9orf72* hexanucleotide repeat expansion was the most common genetic predisposition to ALS (15%). There were generally minimal differences between SC and CTX samples except for shorter PMI and higher RIN in the SC. More information about this dataset can be accessed via the NYGC website (https://www.nygenome.org/contact/).

### Single cell data

The single cell dataset we used is a publicly available dataset that can be accessed via NCBI’s Gene Expression Omnibus (GEO) from series GSE174332 (https://www.ncbi.nlm.nih.gov/geo/query/acc.cgi?acc=GSE174332). At the time the data was accessed for analysis (summer of 2023), there were 23 ALS patients. The dataset involved collecting the nuclei of about 25,000 cells from the primary motor cortex of ALS patients.

To analyze this data, we filtered low-quality cells by removing cells with a proportion of mitochondria-associated genes greater than 0.2, less than 500 total features, less than 500 total unique molecular identifiers (UMIs), and less than 0.8 log_10_ features per UMI. Cell-type classification using ACTIONet [36] had already been performed on the data when accessed, so these cell-type classifications were used in our analysis. We grouped cells into 8 “pseudo-bulk” groups: Layer 5b upper motor neurons, Layer 5/6 excitatory neurons, Layer 2/3 excitatory cells, inhibitory neurons, astrocytes, oligodendrocytes, microglia, and other cells (mostly oligodendrocyte precursor cells with the remaining cell types including fibroblasts, mural cells, and endothelial cells). In order to determine which cell types were most represented in our NMF analysis based on the transcriptional patterns present in the single cell dataset, we utilized Expression Weighted Celltype Enrichment (EWCE) [12].

### ALS subgroup designation

ALS subgroup designations were made based on cluster-specific IPA patterns as well as cell-type-specific transcriptional changes as described in the results section. For example, based on IPA, ALS clusters could be clearly separated in terms of patterns of up- or downregulation of the top 10 most significantly dysregulated pathways: regeneration clusters generally exhibited strong upregulation while degeneration clusters exhibited strong downregulation, and synaptic dysfunction clusters had an intermediate pattern.

### Bulk RNA sequencing data analysis

Details of the analysis are provided in the supplementary methods section of the supplemental information file.

## Supplementary information

Supplemental Material

Supplemental Data

Supplemental Data

Supplemental Data

Supplemental Data

## Data Availability

This research was supported by the Intramural Research Program (NS003130) of the National Institutes of Health (NIH), National Institute of Neurological Disorders and Stroke (NINDS). The contributions of the NIH authors were made as part of their official duties as NIH federal employees, are in compliance with agency policy requirements, and are considered Works of the United States Government. However, the findings and conclusions presented in this paper are those of the author(s) and do not necessarily reflect the views of the NIH or the U.S. Department of Health and Human Services.

## Code availability

Code is available on the following GitHub repository: nickap5/Machine-learning-predicts-distinct-biotypes-ofamyotrophic-lateral-sclerosis.
