# Supplementary material for: Machine learning predicts distinct biotypes of amyotrophic lateral sclerosis
Source: Eur J Hum Genet. 2025 Aug 7;33(10):1290–9. doi: 10.1038/s41431-025-01920-y (PMC12479739; doi:10.1038/s41431-025-01920-y)
Supplement: Supplementary file 1 — Supplemental Material [file 41431_2025_1920_MOESM1_ESM.docx]

Supplementary Materials for

**Machine Learning Predicts Biologically Distinct Subpopulations of Amyotrophic Lateral Sclerosis**

Nicholas Pasternack *et al.*

*Avindra Nath. Email: avindra.nath@nih.gov

**This PDF file includes:**

Supplementary Methods

Supplementary Figures 1-8

Supplementary Tables 1-3

Supplementary Acknowledgements

**Other Supplementary Materials for this manuscript include the following:**

Supplementary Data 1-4

Supplementary Methods

NMF Input

To determine whether there were any associations between NMF cluster designation and metadata variable, Kendall’s τ was calculated for relevant metadata variables and NMF cluster designations. Kendall’s τ was used because of the variability in the scale of variable encoding. The most significant association was between the participating site “academic medical center” and SC_ALSC NMF subgroup (τ = 0.33, p-adj = 1.3E-12). All other associations had an effect size ~ 0.2 or less. It was decided not to remove features associated with site or RIN due to the variability in how these features were represented on the metadata file, low effect size of the associations, associations of these features with each other, and to keep the input to NMF as unbiased as possible.

For the estimation process, 10 runs per cluster (k) from k = 2 through k = 10 were performed setting the random seed to 123211. For the actual clustering, the value of k was set to the optimum value as described above and 500 runs were performed with the random seed set to 123211.

Random Forest Classification

Since the metadata features were mostly categorical and not continuous, they were one-hot encoded using the Caret “dummyVars” function in R. For example, the categorical variable of biological sex (Male or Female) was converted to two variables (Sex_Male and Sex_Female) with a value of 0 (False), and 1 (True). The data was scaled using a MinMax scaler from scikit learn so all features, including continuous ones, were on a scale of 0 to 1.

Since there are multiple NMF classes (three in ALS and four in ALSC analyses), RFC was applied in the context of a One-vs-Rest (OvR) strategy. Essentially, one classifier is fitted per class (e.g. NMF1 vs other three NMF’s). Unless otherwise noted, performance metrics were averaged using the “micro” method (i.e. calculate overall performance metrics using values from all classes such as total TP’s, TN’s, etc.). Feature importance was calculated using the permutation method, with 10 repetitions. The equation for permutation importance where i is feature importance, f is feature, s is score, and R is repetition is:

$$i_{f}=s-(\frac{\sum_{R=1}^{R} s_{R,f}}{R})$$

**SUPPLEMENTARY RESULTS**

<- Supplementary Figure 1. Determination of NMF cluster number.

Data from this figure is based on the ALS patients only NMF in the cortex. The top 5,000 features based on median absolute deviation (MAD) were selected and the variance-stabilizing transformed (VST) abundances of those features were used to cluster the samples. The first metric used to estimate the optimal cluster number is the cophenetic coefficient (A) which relates to the dispersion of the consensus matrix. The local maximum is selected (k = 3 in this case) which is indicated by the arrow. The next method used was the silhouette width, which has a similar interpretation to the cophenetic coefficient, and was plotted for a few different metrics (B). In this case, one might have chosen k = 2 based solely on the silhouette width for the basis metric; however, the other metrics all indicate the optimal number of clusters is k = 3. This can be confirmed by looking at the consensus matrix (C) which shows three distinct clusters. The main area of the consensus matrix represents whether samples are highly distinct (blue) or highly connected (red) based on the clustering with column annotations for basis (the feature matrix, F, which relates to transcript expression), consensus clustering, and silhouette width.


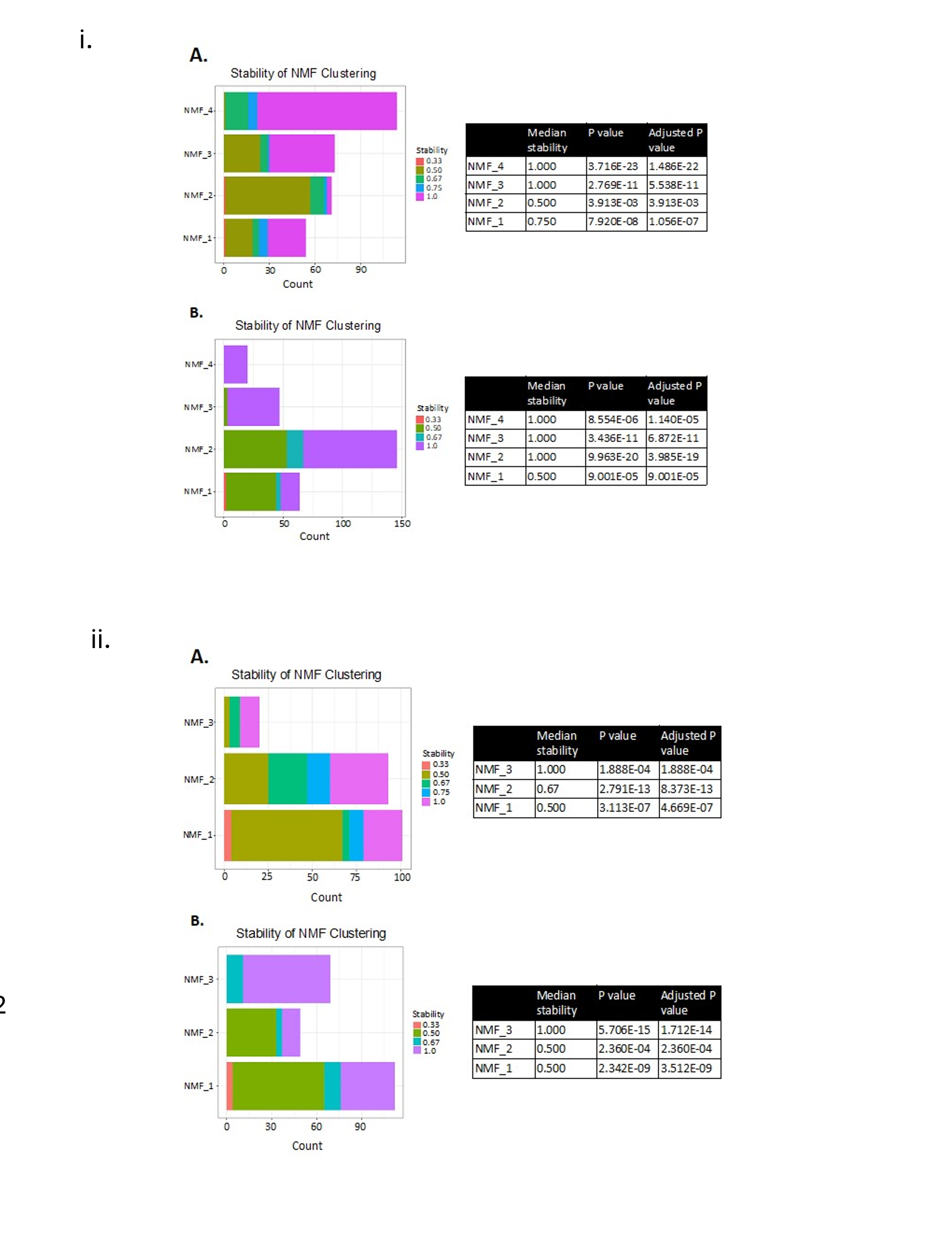


<- **Supplementary Figure 2.** Stability of NMF clustering.

Bar graphs show the number of samples with a given stability (x-axis) for each NMF cluster (y-axis) (left). Table shows P values from a Wilcoxon test for each cluster compared to a μ of 1/median (right) for ALS and control (ALSC) analysis (i) and ALS only (ALS) (ii). The results for (A) cortex and (B) spinal cord are shown for both analysis types. Every NMF cluster has significantly greater stability than would be expected by chance, indicating samples from different CNS subregions from the same patient tend to cluster together.


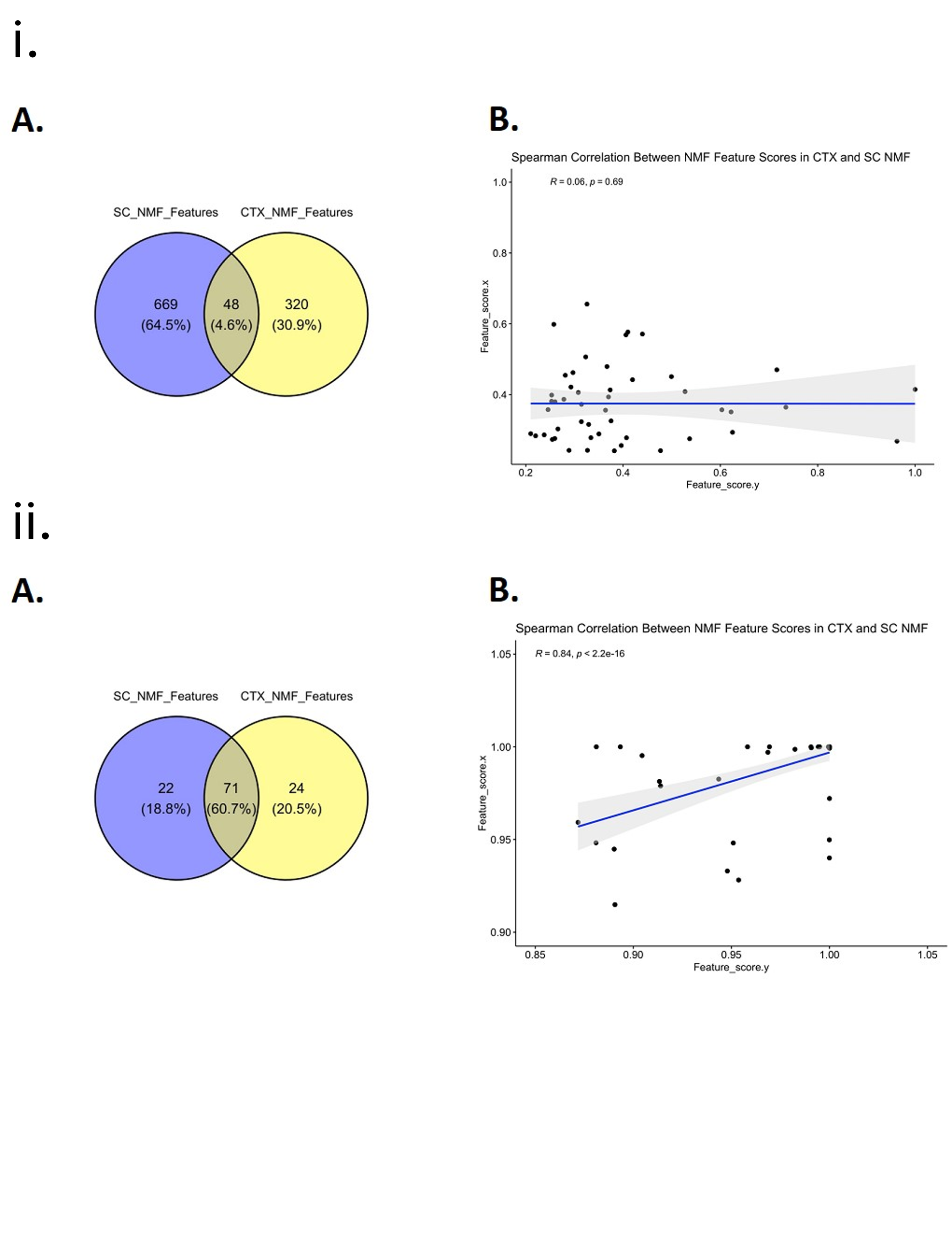


<- **Supplementary Figure 3.** Overlap between selected features across CNS regions.

The NMF selected features from the ALS and control (ALSC) (i) and ALS only (ALS) (ii) analyses were compared. Venn diagram showing percentage and number of overlapping and distinct selected features in cortex (CTX) and spinal cord (SC) NMF analyses (A) and spearman correlation of selected feature scores for the 48 features that overlapped (B). The selected features in the ALS analysis overlapped and their feature scores correlated significantly between the CTX and SC.

**Supplementary Figure 4.** HERV-K coding loci in ALS and controls NMF DEA cortex.

X-axes are significant (DESeq2 FDR adjusted p-value < 0.05). HERV-K genes in green can encode for partial or full-length envelope (Env) protein. Y-axes are differential expression analysis (DEA) log2FC values and color of dot indicates significance (darker is more significant). Feature plots are shown for NMF DEAs comparing NMF 1 (A), 2 (B), 3 (C), and 4 (D) to the other three NMF clusters.

**Supplementary Figure 5.** HERV-K coding loci in ALS and controls NMF DEA spinal cord.

X-axes are significant (DESeq2 FDR adjusted p-value < 0.05) HERV-K genes in green can encode for partial or full-length envelope (Env) protein. Y-axes are differential expression analysis (DEA) log2FC values and color of dot indicates significance (darker is more significant). Feature plots are shown for NMF DEAs comparing NMF 1 (A), 2 (B), 3 (C), and 4 (D) to the other three NMF clusters.

**Supplementary Figure 6.** HERV-K coding loci in ALS patients only NMF DEA cortex.

X-axes are significant (DESeq2 FDR adjusted p-value < 0.05) HERV-K genes in green can encode for partial or full-length envelope (Env) protein. Y-axes are differential expression analysis (DEA)log2FC values and color of dot indicates significance (darker is more significant). Feature plots are shown for NMF DEAs comparing NMF 1 (A), 2 (B), and 3 (C) to the other three NMF clusters.

**Supplementary Figure 7.** HERV-K coding loci in ALS patients only NMF DEA spinal cord.

X-axes are significant (DESeq2 FDR adjusted p-value < 0.05) HERV-K genes in green can encode for partial or full-length envelope (Env) protein. Y-axes are differential expression analysis (DEA)log2FC values and color of dot indicates significance (darker is more significant). Feature plots are shown for NMF DEAs comparing NMF 1 (A), 2 (B), and 3 (C) to the other three NMF clusters.


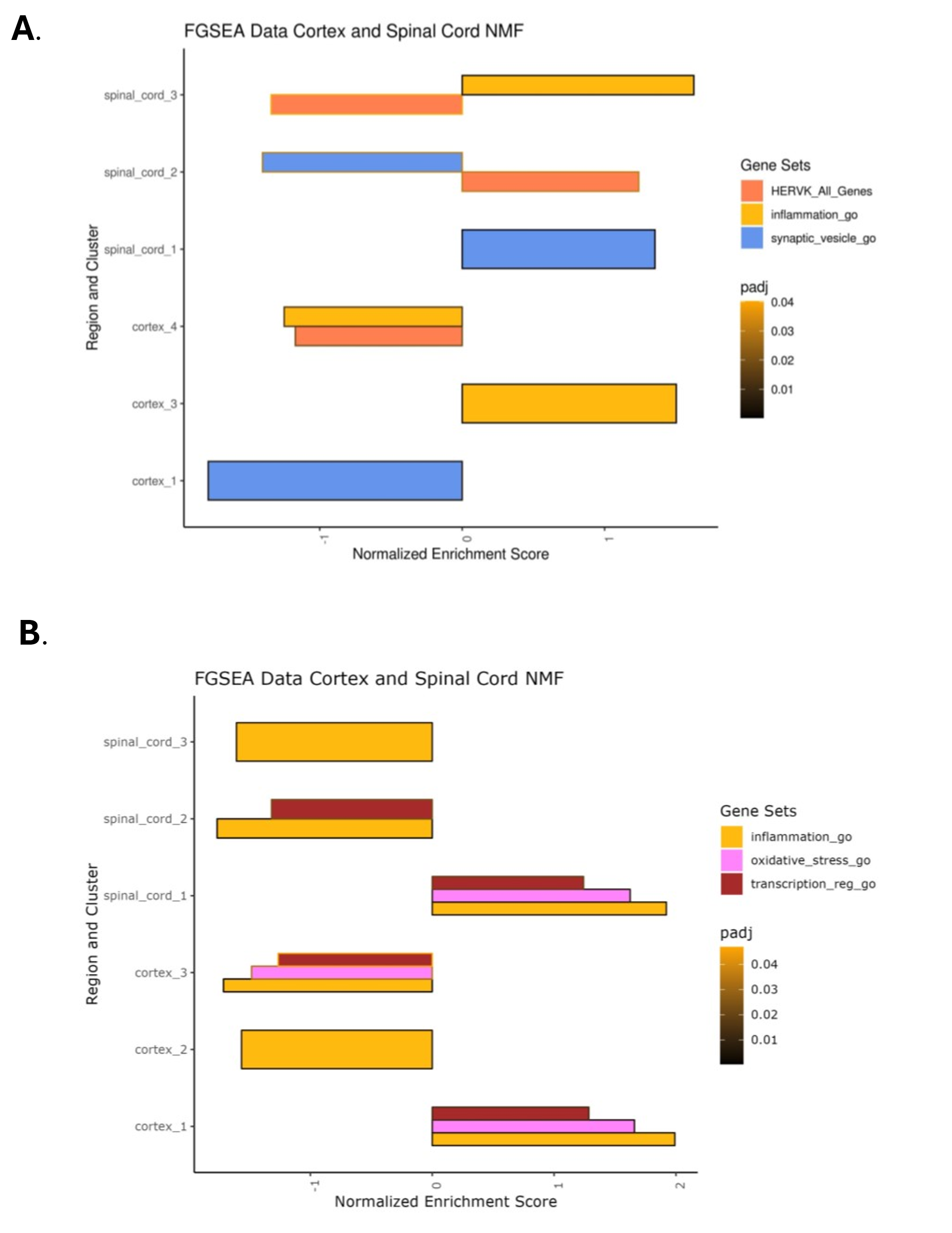


<- Supplementary Figure 8. GSEA for selected gene sets of NMF analyses.

Gene set enrichment analysis (GSEA) was performed for the ALS patients and control (ALSC) analysis (A) and ALS only (ALS) analysis (B). Plots show normalized enrichment score (NES) (x-axis) across NMF clusters (y-axis) in both cortex (CTX) and spinal cord (SC) (same plot) NMF analyses. Only significantly up- or down-regulated gene sets (FDR adjusted GSEA p-value < 0.05) are shown. Color of bar indicates gene set studied and the outline of the bar indicates significance level (darker outline indicates higher significance). Both custom HERV-K and pre-existing GOs were used in the GSEA. Different patterns of significant up- or down-regulation of the custom all HERV-K gene set as well as inflammation- and synapse-related GO gene sets were identified across clusters in both CTX and SC.

**Supplementary Table 1.** Full-length HERV-K Env loci that were dysregulated in at least one of the NMF DEAs.

Footnote: Letter in parentheses after NMF cluster ID represents pathophysiological category: neuronal regeneration (R), synaptic dysfunction (SD), neuronal degeneration (D).

Additional Abbreviations: HERVd ID = ID based on HERVd database nomenclature, CTX = cortex, SC = spinal cord, ALSC = ALS and controls, ALS = ALS only, LTR = Long terminal repeat.

**Supplementary Table 2.** Effect of imputation method on underlying correlations in cortex.

Footnote: Values are shown for the imputed (using predictive mean matching (PMM)) condition (left) and the original raw data condition (right). Rows are sorted based on significance in imputed condition. The values with and without PMM were identical or very similar in all cases. The only meta data variable tested with a significant association with NMF clustering was disease duration.

Supplementary Table 3. Effect of imputation method on underlying correlations in spinal cord.

Footnote: Values are shown for the imputed (using predictive mean matching (PMM)) condition (left) and the original raw data condition (right). Rows are sorted based on significance in imputed condition. The values with and without PMM were identical or very similar in all cases. The meta data variables tested with a significant association with NMF were related NYGC provided metrics of ethnicity.


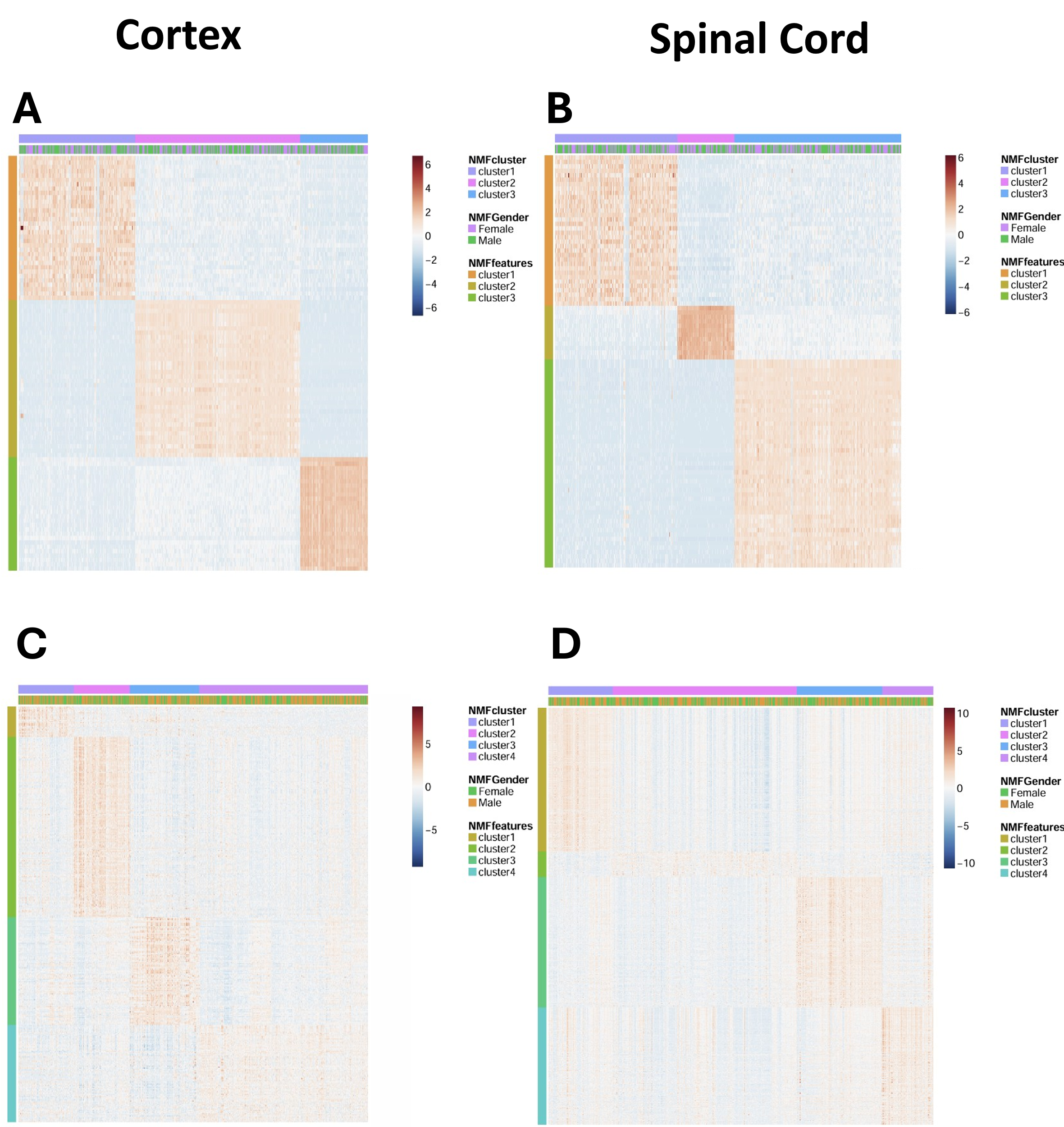


Supplementary Figure 9**.** Heatmap of NMF clusters.

Heatmap was generated using complete hierarchical clustering scaled by rows with column annotations for NMF cluster and biological sex and row annotations for selected features. **(A)** Cortex from ALS patientsshowing three transcriptionally distinct clusters. There is no clear association of biological sex with NMF. **(B)** Spinal cord from ALS patients. Shows three transcriptionally distinct clusters. There is no clear association of biological sex with NMF clustering **(C)** Cortex from ALS patients and controlsshows four transcriptionally distinct clustersThere is no clear association of biological sex with NMF. **(D)** Spinal cord from ALS and controlsshows four transcriptionally distinct clusters. There is no clear association of biological sex with NMF clusters.


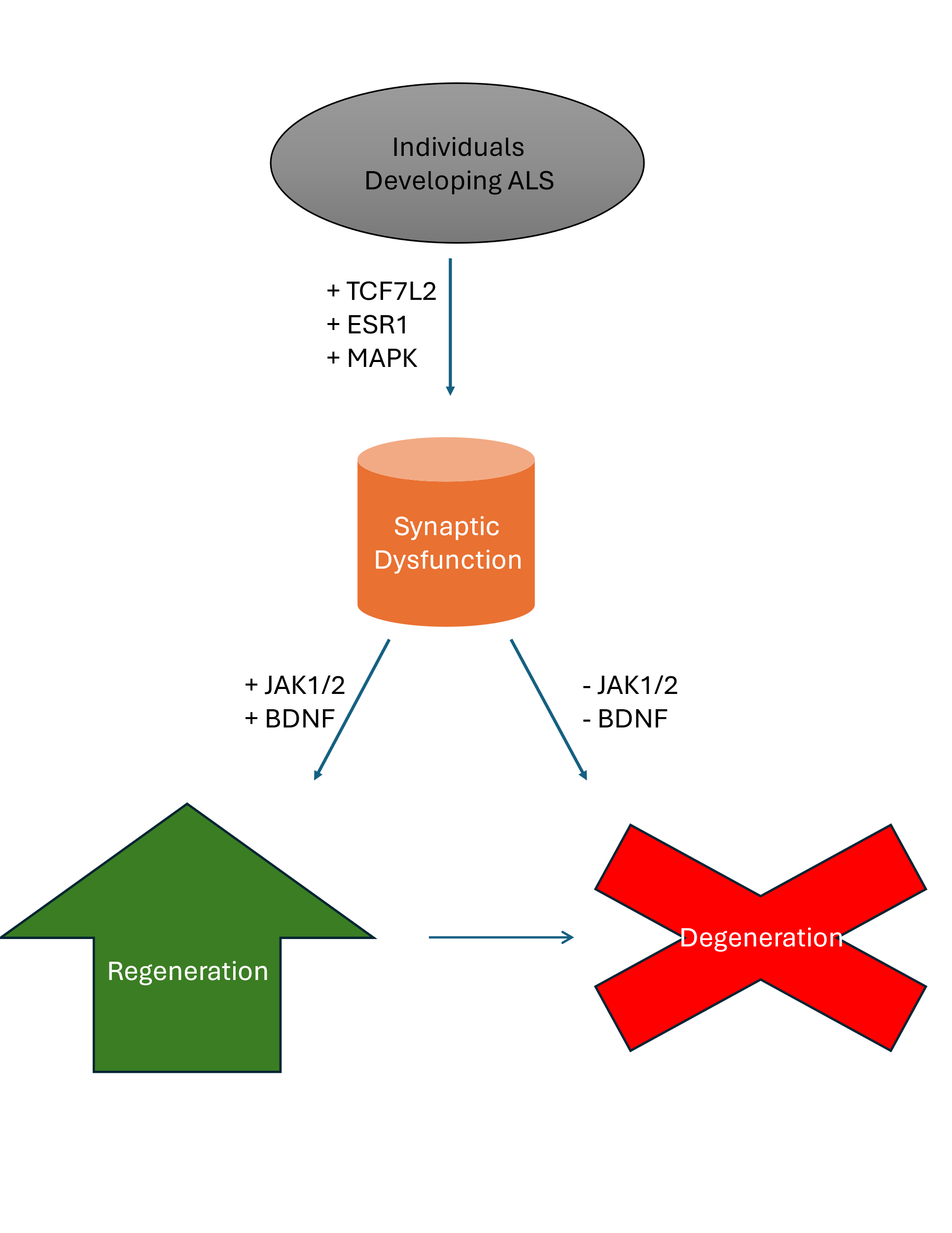


Supplementary Figure 10. Predicted roles of key transcriptional regulators in the pathogenesis of the three ALS biotypes.

Shows the proposed pathogenesis and progression of the three ALS biotypes characterized in this study: synaptic dysfunction, neuronal regeneration, and neuronal degeneration. The synaptic dysfunction biotype may develop first from individuals susceptible to developing ALS (top) under the transcriptional regulation of Transcription factor 7-like 2 (TCF7L2), Estrogen Receptor 1 (ESR1), and mitogen-activated protein kinase (MAPK). At this stage (middle), activation of brain-derived neurotrophic factor (BDNF) and Janus Kinase 1/2 (JAK1/2) drive the neuronal regeneration biotype, while inhibition of these regulators drives the neuronal degermation biotype (bottom).

| **NMF ID** | **Average Age at Death (± SD)** | **Number of Samples (% Total)** | **Female (%)** | **Male (%)** | **ALS (%)** | **C9Orf Pos (%)** |
| --- | --- | --- | --- | --- | --- | --- |
| CTX_ALS_1 | 66 (±10) | 218 (34%) | 48 | 52 | 100 | 13 |
| CTX_ALS_2 | 64 (±10) | 308 (47%) | 46 | 54 | 100 | 14 |
| CTX_ALS_3 | 66 (±10) | 127 (19%) | 48 | 52 | 100 | 11 |
| SC_ALS_1 | 64 (±11) | 193 (35%) | 46 | 54 | 100 | 11 |
| SC_ALS_2 | 66 (±11) | 90 (17%) | 47 | 53 | 100 | 8 |
| SC_ALS_3 | 65 (±10) | 263 (48%) | 49 | 51 | 100 | 15 |
| CTX_ALSC_1 | 64 (±12) | 147 (16%) | 48 | 52 | 73 | 16 |
| CTX_ALSC_2 | 64 (±11) | 148 (16%) | 52 | 48 | 83 | 16 |
| CTX_ALSC_3 | 68 (±10) | 183 (20%) | 42 | 58 | 31 | 11 |
| CTX_ALSC_4 | 65 (±11) | 445 (48%) | 43 | 57 | 82 | 10 |
| SC_ALSC_1 | 63 (±12) | 112 (17%) | 47 | 53 | 73 | 5 |
| SC_ALSC_2 | 65 (±11) | 321 (48%) | 45 | 55 | 88 | 12 |
| SC_ALSC_3 | 63 (±12) | 148 (22%) | 48 | 52 | 91 | 16 |
| SC_ALSC_4 | 69 (±11) | 89 (13%) | 52 | 48 | 54 | 4 |

**Supplementary Table 4.** Demographics of each of the NMF clusters

CTX = cortex; SC = spinal cord; ALS = ALS patients only; ALSC = ALS patients and controls

**SUPPLEMENTAL METHODS**

**Non-negative matrix factorization**

Non-negative matrix factorization (NMF) is an unsupervised algorithm for matrix decomposition used to identify meaningful clusters of samples based on their transcriptional patterns. The optimum cluster number was determined primarily by looking for the local maximum of the cophenetic coefficient ^1^ and the silhouette width ^2^. An example of the process to determine NMF cluster number is highlighted in Supplementary Figure 1.

R scripts used to perform the NMF analysis and subsequent PCA, DEA, and heatmaps were modified from the single-cell RNA-seq analysis and klustering evaluation (SAKE) algorithm ^3^ with permission from the authors. The general equation of NMF, in this application, where M is the original matrix of transcript abundance measures with g genes and p samples; F is a matrix with g genes and k clusters; and C is a matrix of k clusters and p samples, is:

$$M_{g,p}\approx F_{g,k} \times C_{k,p}$$

Beyond the tools in SAKE, a measurement of the robustness of the NMF clustering to individual samples called stability was implemented for both ALS only (ALS) and ALS and controls (ALSC) NMF analyses. The stability metric leverages the fact that multiple samples can come from the same subject in both the cortex (CTX) and spinal cord (SC). Stability is calculated by dividing the number of samples provided by a subject in each cluster by all samples provided by the individual across all clusters. A stability of 1 indicates that all samples derived from the same individual in a CNS region (i.e. CTX or SC) were assigned to the same cluster. This is represented by this formula, where $\sigma$ is the stability for subject P, n is the number of samples, i is the cluster number, and N is the total number of clusters:

$$\sigma_{P}= \frac{n_{i,P}}{\sum_{i=1}^{N} n_{P}}$$

One-sample Wilcoxon signed rank test was performed with μ = 1/median number of samples per patient. In both the CTX and SC, μ = 0.5, median = 2 and the mean was between 2 and 3. Each of the fourteen clusters (four in CTX and SC for ALSC analysis and three in CTX and SC in ALS analysis) had a significantly higher stability than would be expected by chance even when accounting for multiple comparisons using FDR (Supplementary Figure 2). In general, the ALSC analysis had a high median stability (at least 75%) for three of the four NMF clusters, whereas the ALS analysis had a cluster with high median stability in one of three clusters. However, there was variability in terms of stability between NMF clusters: CTX_ALSC_2 and SC_ALSC_1 in the SC both had the lowest median stability (50%) for their respective CNS regions, while CTX_ALS_1 and SC_ALS_1 and SC_ALS_2 had the lowest median stabilities in the ALS analysis (50%). Interestingly, these were generally not the largest clusters in their respective analysis, so the lower stability is not due to a greater number of samples (Supplementary Figure 2).

**Random Forest Classification**

Random forest classification (RFC) was applied to predict NMF cluster based on sample demographics/phenotype. The variables used were biological sex, ethnicity, patient status, family history of ALS or frontotemporal dementia (FTD), FTD with motor neurone disease (MND), MND with dementia, site of motor symptom onset, genetic mutations, *C9ORF72* and *ATXN2* status, El Escorial criteria, comorbidities, age at symptom onset, disease duration (months), *C9ORF72* repeat size, *ATXN2* repeat size, and percent African, South Asian, East Asian, Americas, and European (provided by NYGC). These metadata features were selected since they were included in many entries of the NYGC metadata file and are applicable in a clinical setting.

For the continuous variables, missing data was imputed using predictive mean matching (PMM) ^4^ via the mice function of the Multivariate Imputation by Chained Equations package in R ^5^. The function was run with 5 donor values, 50 maximum iterations, and a random seed = 1. In brief, one randomly selected value is selected from a set of 5 donor values drawn from complete cases of the same feature which are closest to the value predicted for the missing case by a linear model conditional on all other continuous variables.The percent missingness, P, was calculated as below where m is missing cases and t is total cases:

$$P= \frac{m}{t} \times100\%$$

The missingness of the continuous variables are: 36% for age at symptom onset, 31% for disease duration, 22% for *C9ORF72* and *ATXN2* repeat size, and 1% for all percent ethnicities. Therefore, PMM is appropriate for this case.

To further validate this method, Kendall’s tau associations were calculated for each continuous feature imputed with PMM and NMF subtype. In both the CTX (Supplementary Table 2) and SC (Supplementary Table 3), the effect size and significance of the associations were very similar in the PMM and non-PMM conditions. Thus, PMM does not create new or appreciably change pre-existing associations with NMF in the data used for this study. Disease duration (in CTX) and percent European, African, South Asian, and Americas (in SC) were significantly associated with NMF clustering.

Adaptive synthetic sampling (ADASYN) ^6^ was used to ensure a similar number of samples in each cluster for this analysis since there are different numbers of samples in each cluster. The specific implementation of adaptive synthetic sampling used for this analysis was the imbalanced-learn package in Python. The general formula for ADASYN, where s is the number of synthetic data samples that need to be generated for number of minority class n_m_, given the total number of synthetic data samples (S) for the minority class, and density distribution d is:

$$s_{i}=\left( \frac{d_{i}}{\sum_{i=1}^{n_{m}} d_{i}} \right) \times S$$

Scikit learn ^7^ in Python was used to generate the RFC model. The train/test split was 80/20. Prior to training the RFC, five-fold, cross-validated randomized search was performed to tune the number of estimators and maximum depth hyperparameters. The other hyperparameters were not changed from their defaults.

**Pathway and Gene Set Analysis**

Pathway analysis was performed using QIAGEN IPA (<https://digitalinsights.qiagen.com/IPA>). IPA’s canonical pathways and causal network analyses were used to analyze biological pathways and identify potential druggable or diagnostic targets ^8^. The top 10 most dysregulated pathways, according to the sum of the absolute value of the Z-score across rows (i.e. per IPA pathway), are displayed in the relevant heatmaps. Pathways/regulators are displayed in alphabetical order.

The fast gene set enrichment analysis (FGSEA) implementation in R ^9^ was used to assess the degree and significance of gene sets not addressed in IPA, such as those involving HERV-K. The gene sets used include all HERV-K genes, HERV-K genes that encode for partial or full envelope (Env), and HML-2 full-length Env-coding loci. Additional gene sets were derived from the gene ontology (GO) tool AmiGO ^10^ and were accessed using the biomaRt package in R ^11^. The GOs included in this analysis were synaptic vesicle (GO:0008021), neuronal apoptosis (GO:0051402), neuronal death (GO:1901214), motor neuronal death (GO:0097049), presynaptic active zone (GO:0048786), postsynaptic membrane (GO:0045211), gliogenesis (GO:0060252), oxidative stress (GO: 0006979), transcriptional regulation (GO:0140110), and inflammation (GO:0006954) GOs.

The order of the genes used for GSEA was determined by the DEA rank value shown below, where R is the rank, FC is the fold change, and pval is the unadjusted p-value is:

$$R= \left| {log}_{2}FC \right|*-{log}_{10}(pval)$$

**Statistical Analysis**

R versions 3.6.1 and 4.2.2 were used for statistical analysis on the NIH Biowulf high performance computing (HPC) cluster (http://hpc.nih.gov). Details for each analysis type are outlined in the methods section and in the appropriate sections of the main manuscript.

References:

1 Brunet J-P, Tamayo P, Golub TR, Mesirov JP. Metagenes and molecular pattern discovery using matrix factorization. *Proceedings of the National Academy of Sciences* 2004; 101: 4164–4169.

2 Rousseeuw PJ. Silhouettes: A graphical aid to the interpretation and validation of cluster analysis. *J Comput Appl Math* 1987; 20: 53–65.

3 Ho YJ *et al.* Single-cell RNA-seq analysis identifies markers of resistance to targeted BRAF inhibitors in melanoma cell populations. *Genome Res* 2018; 28: 1353–1363.

4 Little RJA. Missing-Data Adjustments in Large Surveys. *Journal of Business & Economic Statistics* 1988; 6: 287.

5 Buuren S van, Groothuis-Oudshoorn K. mice : Multivariate Imputation by Chained Equations in R. *J Stat Softw* 2011; 45. doi:10.18637/jss.v045.i03.

6 Haibo He, Yang Bai, Garcia EA, Shutao Li. ADASYN: Adaptive synthetic sampling approach for imbalanced learning. In: *2008 IEEE International Joint Conference on Neural Networks (IEEE World Congress on Computational Intelligence)*. IEEE, 2008, pp 1322–1328.

7 Pedregosa F *et al.* Scikit-learn: Machine Learning in Python. 2018.

8 Krämer A, Green J, Pollard J, Tugendreich S. Causal analysis approaches in Ingenuity Pathway Analysis. *Bioinformatics* 2014; 30: 523–530.

9 Korotkevich G, Sukhov V, Budin N, Shpak B, Artyomov M, Sergushichev A. Fast gene set enrichment analysis. *bioRxiv* 2016; 17.

10 Carbon S, Ireland A, Mungall CJ, Shu S, Marshall B, Lewis S. AmiGO: online access to ontology and annotation data. *Bioinformatics* 2009; 25: 288–289.

11 Durinck S, Spellman PT, Birney E, Huber W. Mapping identifiers for the integration of genomic datasets with the R/Bioconductor package biomaRt. *Nat Protoc* 2009; 4: 1184–1191.

**SUPPLEMENTARY ACKNOWLEDGEMENTS**

**The NYGC ALS Consortium**

1. Hemali Phatnani, Center for Genomics of Neurodegenerative Disease (CGND), New York Genome Center, New York, NY
2. Justin Kwan, Director of Neurodegeneration Disorders Clinic in the Office of the Clinical Director at the National Institutes of Health, Bethesda, MD
3. Dhruv Sareen, Cedars-Sinai Department of Biomedical Sciences, Board of Governors Regenerative Medicine Institute and Brain Program, Cedars-Sinai Medical Center, and Department of Medicine, University of California, Los Angeles, CA
4. James R. Broach, Department of Biochemistry and Molecular Biology, Penn State Institute for Personalized Medicine, The Pennsylvania State University, Hershey, PA
5. Zachary Simmons, Department of Neurology, The Pennsylvania State University, Hershey, PA
6. Ximena Arcila-Londono, Department of Neurology, Henry Ford Health System, Detroit, MI
7. Edward B. Lee, MD, PhD, Department of Pathology and Laboratory Medicine, Perelman School of Medicine, University of Pennsylvania, Philadelphia, PA
8. Vivianna M. Van Deerlin, Department of Pathology and Laboratory Medicine, Perelman School of Medicine, University of Pennsylvania, Philadelphia, PA
9. Neil A. Shneider, MD, Department of Neurology, Center for Motor Neuron Biology and Disease, Institute for Genomic Medicine, Columbia University, New York, NY
10. Ernest Fraenkel, Department of Biological Engineering, Massachusetts Institute of Technology, Cambridge, MA
11. Lyle W. Ostrow, MD, Department of Neurology, Temple University Lewis Katz School of Medicine, Philadelphia, PN
12. Frank Baas, Department of Neurogenetics, Academic Medical Centre, Amsterdam and Leiden University Medical Center, Leiden, The Netherlands
13. Noah Zaitlen, Computational and Medical Genomics, University of California, Los Angeles, CA
14. James D. Berry, ALS Multidisciplinary Clinic, Neuromuscular Division, Department of Neurology, Harvard Medical School, and Neurological Clinical Research Institute, Massachusetts General Hospital, Boston, MA
15. Andrea Malaspina, Centre for Neuroscience and Trauma, Blizard Institute, Barts and The London School of Medicine and Dentistry, Queen Mary University of London, London, and Department of Neurology, Basildon University Hospital, Basildon, United Kingdom
16. Pietro Fratta, MD, Institute of Neurology, National Hospital for Neurology and Neurosurgery, University College London, London, United Kingdom
17. Gregory A. Cox, The Jackson Laboratory, Bar Harbor, ME
18. Leslie M. Thompson, Department of Psychiatry & Human Behavior, Department of Biological Chemistry, School of Medicine, and Department of Neurobiology and Behavior, School of Biological Sciences, University California, Irvine, CA
19. Steve Finkbeiner, Taube/Koret Center for Neurodegenerative Disease Research, Roddenberry Center for Stem Cell Biology and Medicine, Gladstone Institute
20. Efthimios Dardiotis, Department of Neurology & Sensory Organs, University of Thessaly, Thessaly, Greece
21. Timothy M. Miller, MD, Department of Neurology, Washington University in St. Louis, St. Louis, MO
22. Siddharthan Chandran, Centre for Clinical Brain Sciences, Anne Rowling Regenerative Neurology Clinic, Euan MacDonald Centre for Motor Neurone Disease Research, University of Edinburgh, Edinburgh, United Kingdom
23. Suvankar Pal, Centre for Clinical Brain Sciences, Anne Rowling Regenerative Neurology Clinic, Euan MacDonald Centre for Motor Neurone Disease Research, University of Edinburgh, Edinburgh, United Kingdom
24. Eran Hornstein, Department of Molecular Genetics, Weizmann Institute of Science, Rehovot, Israel
25. Daniel J. MacGowan, Department of Neurology, Icahn School of Medicine at Mount Sinai, New York, NY
26. Terry Heiman-Patterson, Center for Neurodegenerative Disorders, Department of Neurology, the Lewis Katz School of Medicine, Temple University, Philadelphia, PA
27. Molly G. Hammell, New York University, Institute of Systems Genetics, Department of Neuroscience and Physiology, New York, NY
28. Nikolaos. A. Patsopoulos, Computer Science and Systems Biology Program, Ann Romney Center for Neurological Diseases, Department of Neurology and Division of Genetics in Department of Medicine, Brigham and Women’s Hospital, Boston, MA, Harvard Medical School, Boston, MA, and Program in Medical and Population Genetics, Broad Institute, Cambridge, MA
29. Oleg Butovsky, Ann Romney Center for Neurologic Diseases, Brigham and Women's Hospital, Harvard Medical School, Boston, MA
30. Joshua Dubnau, Department of Anesthesiology, Stony Brook University, Stony Brook, NY
31. Robert Bowser, Department of Neurology, Barrow Neurological Institute, St. Joseph's Hospital and Medical Center, Department of Neurobiology, Barrow Neurological Institute, St. Joseph's Hospital and Medical Center, Phoenix, AZ
32. Matt Harms, Department of Neurology, Division of Neuromuscular Medicine, Columbia University, New York, NY
33. Eleonora Aronica, Department of Neuropathology, Academic Medical Center, University of Amsterdam, Amsterdam, The Netherlands
34. Mary Poss, DVM, Department of Biology and Veterinary and Biomedical Sciences, The Pennsylvania State University, University Park, PA
35. Jennifer Phillips-Cremins, New York Stem Cell Foundation, Department of Bioengineering, School of Engineering and Applied Sciences, University of Pennsylvania, Philadelphia, PA
36. John Crary, MD, Department of Pathology, Fishberg Department of Neuroscience, Friedman Brain Institute, Ronald M. Loeb Center for Alzheimer's Disease, Icahn School of Medicine at Mount Sinai, New York, NY
37. Nazem Atassi, Department of Neurology, Harvard Medical School, Neurological Clinical Research Institute, Massachusetts General Hospital, Boston, MA
38. Dale J. Lange, Department of Neurology, Hospital for Special Surgery and Weill Cornell Medical Center, New York, NY
39. Darius J. Adams, Medical Genetics, Atlantic Health System, Morristown Medical Center, Morristown, NJ, and Overlook Medical Center, Summit, NJ
40. Leonidas Stefanis, Center of Clinical Research, Experimental Surgery and Translational Research, Biomedical Research Foundation of the Academy of Athens (BRFAA), 4 Soranou Efesiou Street, 11527, Athens, Greece; 1st Department of Neurology, Eginition Hospital, Medical School, National and Kapodistrian University of Athens, Athens, Greece
41. Marc Gotkine, Neuromuscular/EMG service and ALS/Motor Neuron Disease Clinic, Hebrew University-Hadassah Medical Center, Jerusalem, Israel
42. Robert H. Baloh, Board of Governors Regenerative Medicine Institute, Los Angeles, CA; Department of Neurology, Cedars-Sinai Medical Center, Los Angeles, CA
43. Suma Babu, MBBS, Neurological Clinical Research Institute, Massachusetts General Hospital, Boston, MA
44. Towfique Raj, Departments of Neuroscience, and Genetics and Genomic Sciences, Ronald M. Loeb Center for Alzheimer's disease, Icahn School of Medicine at Mount Sinai, New York, NY
45. Sabrina Paganoni, Harvard Medical School, Department of Physical Medicine & Rehabilitation, Spaulding Rehabilitation Hospital, Boston, MA
46. Ophir Shalem, Center for Cellular and Molecular Therapeutics, Children's Hospital of Philadelphia, Philadelphia, PA; Department of Genetics, Perelman School of Medicine, University of Pennsylvania, Philadelphia, PA
47. Colin Smith, Centre for Clinical Brain Sciences, University of Edinburgh, Edinburgh, UK; Euan MacDonald Centre for Motor Neurone Disease Research, University of Edinburgh, Edinburgh, UK
48. Bin Zhang, Department of Genetics and Genomic Sciences, Icahn Institute of Data Science and Genomic Technology, Icahn School of Medicine at Mount Sinai, New York, NY
49. University of Maryland Brain and Tissue Bank and NIH NeuroBioBank
50. Brent Harris, Department of Neuropathology, Georgetown Brain Bank, Georgetown Lombardi Comprehensive Cancer Center, Georgetown University Medical Center, Washington DC
51. Iris Broce, Neuroradiology Section, Department of Radiology and Biomedical Imaging, University of California, San Francisco, San Francisco, CA
52. Vivian Drory, Neuromuscular Diseases Unit, Department of Neurology, Tel Aviv Sourasky Medical Center, Sackler Faculty of Medicine, Tel-Aviv University, Tel-Aviv, Israel
53. John Ravits, Department of Neuroscience, University of California San Diego, La Jolla, CA
54. Corey McMillan, Department of Neurology, University of Pennsylvania Perelman School of Medicine, Philadelphia, PA
55. Vilas Menon, Department of Neurology, Columbia University Medical Center, New York, NY
56. Lani Wu, Department of Pharmaceutical Chemistry, University of California San Francisco, San Francisco, CA
57. Steven Altschuler, Department of Pharmaceutical Chemistry, University of California San Francisco, San Francisco, CA.
